# Impact of the COVID-19 Pandemic on the Management of Gallbladder, Biliary Tract, and Pancreatic Diseases

**DOI:** 10.7759/cureus.43473

**Published:** 2023-08-14

**Authors:** Amnah Ilyas Khan, Christophe Thomas, Hettie O'Connor, Frederick Dowker, Liam Horgan, Murad A Khan

**Affiliations:** 1 General Surgery, Northumbria Healthcare NHS Foundation Trust, Newcastle upon Tyne, GBR; 2 Surgery, Northumbria Healthcare NHS Foundation Trust, Newcastle upon Tyne, GBR; 3 Surgery, Mater Hospital Brisbane, Queensland, AUS; 4 General Surgery, Shifa International Hospital Islamabad, Islamabad, PAK

**Keywords:** pancreatitis, acute cholelithiasis, covid 19, biliary diseases, acutely inflamed gallbladder

## Abstract

Introduction

Biliary diseases are a major acute general surgical burden. Laparoscopic cholecystectomy is the gold standard surgical procedure, although it was discontinued during an outbreak. Effective management permits decisive therapy, symptom alleviation, and fewer hospitalizations and complications. Throughout the initial COVID-19 situation, surgical procedures for patients were delayed. Invasive services were required to employ conservative or non-operative therapy, which could lead to increased recurring presentations and biliary-pancreatic problems.

Aim

Examining the impact of COVID-19 on the outcomes and hospitalizations of patients suffering from gallstone, biliary tract, and pancreatic diseases.

Methods

The retrospective analysis included patients with the following ICD-10 codes who presented to our unit: cholelithiasis (K80), cholecystitis (K81), and acute pancreatitis (K85). We compared the interval of the first COVID-19 pandemic wave, from March to August 2020, with the period before the pandemic, referred to as Pre-COVID-19. After applying exclusion criteria, a total of 868 patients were enrolled in the trial, having initially recruited around 1,400 individuals using these codes. Patients with inaccurate coding, cancer, or non-stone disease were excluded (e.g., alcoholic pancreatitis). The demographic information, admission details, investigations, surgical therapy, operating specifics, and postoperative complications of the patients were noted. Changes in surgical management, patient representation, and postoperative complications were the key outcomes.

Results

A statistically significant (p<0.05) rise was seen in repeat presentations in the COVID group, most likely due to the failure of definitive treatment. The other outcome is the distribution of presentations was comparable, patients with acute cholecystitis and gallstone pancreatitis showed statistically significant (p<0.05) lower rates of definitive therapy.

Conclusion

During the COVID period, all surgeries except those for cancer were halted. Unknown causes led to several consequences related to the gallbladder, biliary tract, and pancreas. Patients with cholecystitis, gallstone pancreatitis, and pancreatic inflammation experienced a lower probability of treatment. The increase in hospitalizations and self-presentations indicated that definitive therapy, designed to restrict COVID-19 exposure, actually increased patient risk. Despite this risk, we had no COVID-19 instances in our cohort. The evaluation of the long-term consequences of the pandemic on acute pancreatitis and its care will require a large-scale, multicenter investigation.

## Introduction

The COVID-19 pandemic has prompted a global rearrangement of medical services. During the initial phase of the pandemic, elective procedures not related to cancer were halted. Several studies have demonstrated a drop in the surgical procedures implemented during COVID-19; nevertheless, there have been varying reports regarding the severity of the cases. As the epidemic evolved, a better understanding of COVID emerged, guiding the diagnosis and management of patients needing surgery. However, due to the pandemic situation, there was a delay in surgeries, which could compromise patient outcomes [1-4]. Acute pancreatitis is defined by inflammation of the pancreas, loss of acinar cells, and either local or systemic effects [5]. The COVID-19 investigation revealed a year of worse outcomes in patients with gallstone pancreatitis and acute cholecystitis who were also affected by COVID-19. These outcomes included increased fatality rates, more severe cases of pancreatitis and cholecystitis, longer hospital stays, and organ failure [6-8]. Cholelithiasis is a worldwide condition that remains a primary reason for operating procedures, accounting for a substantial part of healthcare costs [9]. Current prevalence rates for cholelithiasis range from 10 to 15 percent in Western countries and 3 to 4 percent in Asian cultures. According to the report presented by The National Institute for Health and Care Excellence (NICE), every 56 and 5 cases out of a hundred thousand patients are diagnosed with acute and chronic pancreatitis, respectively [10]. One to three percent of patients have the potential to develop complications each year. These repercussions include acute pancreatitis, acute cholecystitis, and cholangitis, all of which may result in numerous hospitalizations [11]. The treatment of choice for symptomatic gallstones is laparoscopic cholecystectomy, which is considered the gold standard. NICE recommends that cholecystectomy be performed within one week of acute cholecystitis diagnosis and within the same admission for gallstone pancreatitis [12]. Problems with untreated gallstones might lead to a return of the original symptoms and possibly additional hospitalizations. During the time of the outbreak, the Intercollegiate Guidelines suggested cholecystostomy drainage or non-surgical treatment for acute biliary disease [13]. As a direct result, the number of cholecystectomies performed during the outbreak decreased. Similarly, the management of gallstone disease during the COVID-19 pandemic (MEGAVID) clinical investigator group found that the rate of cholecystectomy has decreased by 72.2% since the beginning of the pandemic [2]. This article's primary concern is the direct result of these factors, which leads to an increased risk of readmissions and complications due to gallstone disease.

## Materials and methods

Study design

We performed a retrospective cohort analysis of all consecutive adult patients (older than 18 years) who presented to our high-volume emergency care hospital either via the ED or the Surgical Assessment Unit (SAU) with the following ICD-10 codes: cholelithiasis (K80), cholecystitis (K81), and acute pancreatitis (K85). Patients were divided into two cohorts: Pre-COVID (March 2019-August 2019) and during the COVID-19 pandemic (March 2020-August 2020). The study was approved by the Northumbria Healthcare Foundation Trust's Institutional Review Board (approval no. C4196) for ethical considerations.

Data collection

A digital note review was conducted, and the following information was gathered: patient demographics, admission date, admission location, diagnosis (ICD code), length of stay (LOS), investigations, treatments (conservative/operative), operative variables, and post-op complications (classified by) [14]. All patients' notes were reviewed for presentations and recurrent episodes for six months before and after the first presentation in the study period.

Exclusion criteria

Coding accuracy was assessed; those with inaccurate coding, cancer, and non-stone disease were excluded (e.g., alcoholic pancreatitis).

Definitions

The patients were assessed in the SAU or emergency unit of the hospital. The same patient criteria were used for recruitment regarding the selected variable before the COVID-19 pandemic. Patients who were diagnosed with gallstones and cholecystitis underwent laparoscopic cholecystectomy using a standard four-port approach. Current guidelines advocate early decisive therapy for patients with mild gallstone pancreatitis to prevent recurring attacks. Formal recommendations for early cholecystectomy are supported by several organizations, including the British Society of Gastroenterology (1998), the UK Working Party on Acute Pancreatitis (2005) [14], the Japanese Society of Hepato-Biliary-Pancreatic Surgery (2006) [15], the International Association of Pancreatology (IAP, 2002) [16], and the American Gastroenterological Association (AGA, 2007) [17]. The guidelines issued by the IAP in 2002 suggest the following treatments for mild gallstone pancreatitis: cholecystectomy as soon as the patient has recovered, ideally during the same hospitalization, and endoscopic sphincterotomy as an alternative to cholecystectomy for patients who are not surgical candidates [16]. Data variables include demographics, blood tests, primary diagnosis, surgical or endoscopic techniques for the pseudocyst of the pancreas, and LOS of the same patient admitted pre-COVID and during COVID. In addition, follow-up data were also obtained. Pancreatitis with unclear etiology was described as individuals who did not have a defined etiology during this hospitalization despite repeated investigations at the time of discharge/death.

Outcome measure

Data were assessed with descriptive statistics. Categorical variables were analyzed using the Chi-Square test, and continuous variables were assessed using the Mann-Whitney test, all performed with SPSS version 22.0 (IBM Corp., Armonk, NY, USA).

Aim of the study

Examining the impact of COVID-19 on the outcomes and hospitalizations of patients suffering from gallstone, biliary tract, and pancreatic diseases. 

## Results

In Table 1, the baseline characteristics of the study are presented. Initially, 1400 patients were recruited with this coding, but after applying the exclusion criteria, 863 patients were selected for the study. The total number of patients was 863, of which 409 were enrolled before the COVID pandemic, and 454 patients were enrolled during the COVID pandemic. The unique patients for this study and repeat presentations were also calculated. We also focused on the patients' LOS in days to rule out any difference between the Pre-COVID and during-COVID periods.

**Table 1:** Baseline characteristics. The values for admission details, presentation site, grade, and discharge site are presented as n (%). Length of stay is measured in days and presented as the mean (95% CI), and age is presented as the number of years (95% CI). Descriptive analysis and the Chi-Square test were used to test for significance. A p-value of <0.05 was considered significant. SAU: Surgical assessment unit; LOS: Length of Stay.

Demographics	Pre-COVID	COVID	Total
Age	59.7 (57.7-61.6)	58.6 (56.8-60.4)	
Admission Details			
Total number of patient presentations	409	454	863
Number of new patients in the study	376	319	695
No. of patients with repeat presentations in study period, n (%)	38 (10%)	77 (24.13%)	115 (16.2%)*
No. of repeat presentations, n (%)	33 (9.3%)	135 (40.7%)	170 (24.04%)*
Length of stay (in days) mean (CI)	3.1 (2.5-3.8)	2.5 (1.9-3)	
LOS for primary presentations, n (%)	1254 (59.3%)	859 (40.6%)	
Mean LOS for primary presentations	3.3 (2.6-4)	2.7 (2-3.3)	
Total LOS for repeat presentations	45	286	331
Mean LOS for repeat presentations	1.4 (0.6-2.4)	2.1 (1-3.2)	
Presentation site	408	438	846
ED, n (%)	396 (97.0%)	392 (89.4%)	788
SAU via Primary care, n (%)	12 (2.9)	46 (10.5)	58

The main outcomes are detailed in Table 2. This includes comparisons between the primary diagnosis, antibiotics used, surgery outcomes (such as types and episodes of surgery), and management outcomes, including definitive management for cholecystitis and gallstone pancreatitis before and after COVID. Adverse features and complications were also compared, such as mean operative time, mean blood loss, drain use, post-operation care, intensive therapy unit (ITU) admissions, and length of stay (in days). The duration of antibiotic use in days was also noted. It was clearly seen in the results that in the era of the COVID pandemic, the rate of surgery was significantly reduced, and decreases were observed in laparoscopic cholecystectomy, elective procedures, and procedures conducted for biliary colic as the sole indication. However, the trend of the primary diagnosis, excluding pancreatitis, was non-significant but increased during COVID. It is essential to note that the data presented in the COVID section did not pertain to COVID-positive patients. No significant differences were observed in variables such as main operative time, mean blood loss, drain use, post-operation ITU admission, and ITU LOS.

**Table 2:** Comparison of outcomes between Pre-COVID and COVID periods. The data are presented as count (n) and percentage (%). The Chi-square test was used for categorical variables and the Mann-Whitney U test for continuous variables by using SPSS version 22.0. P-value <0.05 and p-value <0.01 were considered as significant. ITU: Intensive therapy unit; USS: Ultrasound scan; MRCP: Magnetic Resonance Cholangiopancreatography; ERCP: Endoscopic Retrograde Cholangiopancreatography; CBD:  Common bile duct.

Variable	Pre-COVID	COVID	Total	P-value
Primary Diagnosis				NS
Biliary colic	35 (8.56%)	54 (11.89%)	89 (10.3%)	
Cholangitis,	21 (5.13%)	26 (5.73%)	47 (5.4%)	
Calculous Cholecystitis	141 (34.47%)	160 (35.24%)	301 (34.9%)	
Gallstone Pancreatitis	6 (1.47%)	16 (3.52%)	22 (2.5%)	
Gallstones with Cholecystitis	164 (40.10%)	166 (36.56%)	330 (38.2%)	
Pancreatitis	42 (10.27%)	32 (7.05%)	74 (8.6%)	
Diagnostics and antibiotics				NS
Antibiotics	237 (57.95%)	209 (46.4%)	446 (51.68%)	
Antibiotics length (in days), mean	6.4	8.20		
USS	323 (78.97%)	263 (57.93%)	586 (67.9%)	
CT, n (%)	123 (30.07%)	104 (22.91%)	227 (26.3%)	
MRCP	168 (41.08%)	149 (32.82%)	317 (36.7%)	
ERCP, n (%)	75 (18.34%)	64 (14.10%)	139 (16.1%)	
Operative management				<0.0001
Cholecystectomy – laparoscopic	196 (47.92%)	194 (42.73%)	390 (45.19%)	
Cholecystectomy + cholangiogram	9 (2.20%)	1 (0.22%)	10 (1.16%)	
Cholecystectomy + exp. CBD	5 (1.22%)	3 (0.66%)	8 (0.93%)	
Laparoscopic subtotal cholecystectomy	8 (1.96%)	8 (1.76%)	16 (1.85%)	
Laparoscopic subtotal cholecystectomy + cholangiogram	1 (0.24%)	0	1 (0.12%)	
Laparoscopic division of adhesions	0	2 (0.44%)	2 (0.23%)	
Diagnostic Laparoscopy	0	2 (0.44%)	2 (0.23%)	
Episodes of surgery				<0.0001
Elective, n (%)	82 (20.0%)	125 (27.5%)	207 (23.9%)	
Acute episode	123 (30.0%)	64 (14.1%)	187 (21.6%)	
No previous acute episodes	59 (14.4%)	0	59 (6.8%)	
Presentations of gallstone pancreatitis who had index procedure n (%)	42 (80.76%)	34 (53.1%)	76 (65.5%)	
Previous acute episodes	4 (0.9%)	2 (0.4%)	6 (0.7%)	
Previous acute episodes (unique patient in the study)	8 (1.9%)	12 (2.6%)	20 (2.3%)	
Intra-op adverse features or complications	50 (12.2%)	60 (13.2%)	110 (12.7%)	<0.001
Post-operative complications	33 (8.06%)	39 (8.5%)	72 (8.3%)	<0.05
Cholecystitis definitive management				<0.0001
Cholecystitis presentation	305 (74.5%)	326 (71.8%)	631 (73.1%)	
Presentations of cholecystitis who had index procedure n (%)	110 (36.07%)	50 (15.34%)	160 (25.3%)	
Gallstone pancreatitis definitive management				<0.0001
Gallstone pancreatitis presentation	52 (12.71%)	64 (14.09%)	116 (13.4%)	
Presentations of gallstone pancreatitis who had index procedure n (%)	42 (80.76%)	34 (53.1%)	76 (65.5%)	

**Figure 1:**
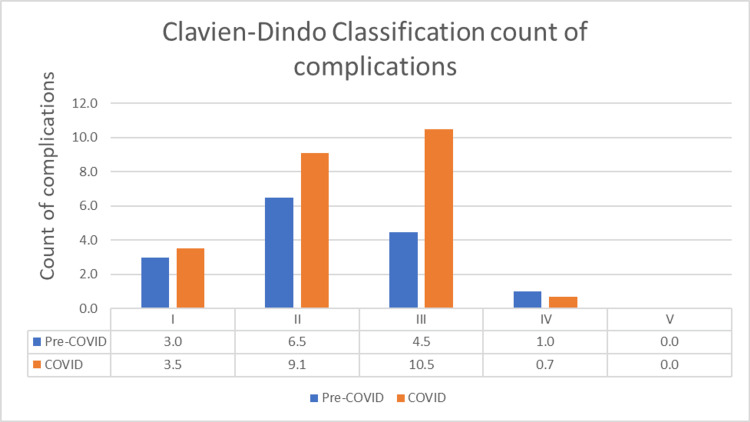
Count of complications according to the Clavien-Dindo classification. The data are presented as percentages in grades (I to V) for both pre-COVID and COVID periods.

In the present research, we included all patients treated with surgery during both periods and evaluated them using the Clavien-Dindo classification to measure postoperative surgical complications. The working group has employed the Clavien-Dindo classification as a tool for recording surgical morbidity within the context of clinical research projects. Complications following laparoscopic cholecystectomy, cholecystectomy with cholangiogram, and laparoscopy were coded and are presented in Figure 1.

## Discussion

Our cohort groups were comparable in age diagnosis of patients with no difference before and after the COVID-19 pandemic (Tables 1-2). The number of surgeries performed during the COVID-19 epidemic for simple gallstone disease decreased significantly (Table 2). The number of patients receiving cholecystectomy declined three times following the decrease in the surgical practices conducted for acute cholecystitis, biliary/ gallstone pancreatitis, and cholangitis. However, there has been an increase in the trend to perform early cholecystectomy due to other causes, such as acute pancreatitis caused by gallstones [18]. In the past era, laparoscopic surgery for gallstone disease has become more prevalent among octogenarians [19]. At the onset of the COVID-19 pandemic, it was anticipated that many surgeries would be canceled due to a shift in priorities [20, 21]. Despite this, the aggregate number of patients requiring elective surgery will likely increase due to the decline in planned procedures during the pandemic. Efforts have been made to meet the demand for gallstone surgery in 2021; however, there are no data on the number of individuals with symptomatic gallstone disease who are still awaiting surgery. During the first year of the epidemic, concerns were raised regarding the transmission of SARS-CoV-2 by aerosols generated in the operating room during laparoscopic procedures [22]. However, later research demonstrated that this concern was exaggerated [23]. Several European surgical organizations advised laparoscopic cholecystectomy for acute cholecystitis, even during the continuing epidemic [24]. During the pandemic, there was a decline in the number of open cholecystectomies performed, but this may have been the end of a long-term trend of abandoning open techniques. Reduced LOS in the patients presented during the COVID-19 pandemic indicated an attempt to decrease patient hospitalization and possibly COVID-19 exposure; however, this difference was not statistically significant. There was a statistically significant rise in the number of repeat presentations in the COVID-19 cohort, and we believe that this is most likely due to the ineffectiveness of conservative therapy. Even though there was a similar distribution of presentations, patients who presented with acute cholecystitis and gallstone pancreatitis had statistically significantly lower rates of definitive management (Table 2). This was the case, although there was no difference in the distribution of presentations. The most likely explanation for the statistically significant rise in poor operative outcomes is that patients are presenting for treatment later and initially opting for conservative care (Figure 1). There is a correlation between the increase in adverse results, the complexity of surgeries, and the failure of operative management. These factors collectively led to an increase in complication rates within the COVID-19 cohort. The goal of conservative therapy was to shield patients from COVID-19 exposure; however, this approach inadvertently heightened risks. As evidenced by the rise in re-presentations and, consequently, hospitalizations and sequelae, the protective strategy may have been counterproductive. During the COVID-19 period, the intent of conservative management is to minimize patient exposure to the virus by avoiding admission and trying to keep beds free for patients who may need urgent care related to COVID-19. 

## Conclusions

The pandemic had a profoundly negative impact on routine surgeries. During this period, a considerable proportion of individuals presented with gallbladder, biliary tract, and pancreas-related outcomes of uncertain cause. Although the two groups were comparable with regard to demographics, the distribution of main diagnoses showed significant variations in outcomes. There is a significant difference in patients who presented with gallstone pancreatitis, cholecystitis, and pancreatic inflammation. A correlation exists between the rise in adverse findings and the increased complexity of operations in the COVID-19 cohort, leading to increased complications. This pathway, however, accidentally led to greater consequences for patients, as evidenced by an increase in the number of patients who either presented themselves or were admitted to the hospital. Despite the risk, our group did not include any individuals who developed COVID-19. Treatment for gallstone pancreatitis and acute cholecystitis should be performed as soon as possible. The surgeons must go laparoscopic method of performing cholecystectomy ought to be regarded as the default method for operating on the vast majority of patients. If COVID-19 numbers rise again in future years, medical units should strive to manage patients in line with guidelines as closely as possible. As we have demonstrated, deviating from guidelines can increase the burden of complications, lead to more challenging procedures, create additional complications, and result in an increase in inpatient stays.
